# A Novel Role for Banana *MaASR* in the Regulation of Flowering Time in Transgenic *Arabidopsis*

**DOI:** 10.1371/journal.pone.0160690

**Published:** 2016-08-03

**Authors:** Peiguang Sun, Hongxia Miao, Xiaomeng Yu, Caihong Jia, Juhua Liu, Jianbin Zhang, Jingyi Wang, Zhuo Wang, Anbang Wang, Biyu Xu, Zhiqiang Jin

**Affiliations:** 1 Haikou Experimental Station, Chinese Academy of Tropical Agricultural Sciences, Haikou 570102, China; 2 Key Laboratory of Tropical Crop Biotechnology, Ministry of Agriculture, Institute of Tropical Bioscience and Biotechnology, Chinese Academy of Tropical Agricultural Sciences, Haikou 571101, China; South China Agricultural University, CHINA

## Abstract

The abscisic acid (ABA)-, stress-, and ripening-induced (ASR) protein is a plant-specific hydrophilic transcriptional factor involved in fruit ripening and the abiotic stress response. To date, there have been no studies on the role of *ASR* genes in delayed flowering time. Here, we found that the *ASR* from banana, designated as *MaASR*, was preferentially expressed in the banana female flowers from the eighth, fourth, and first cluster of the inflorescence. *MaASR* transgenic lines (L14 and L38) had a clear delayed-flowering phenotype. The number of rosette leaves, sepals, and pedicel trichomes in L14 and L38 was greater than in the wild type (WT) under long day (LD) conditions. The period of buds, mid-flowers, and full bloom of L14 and L38 appeared later than the WT. cDNA microarray and quantitative real-time PCR (qRT-PCR) analyses revealed that overexpression of *MaASR* delays flowering through reduced expression of several genes, including photoperiod pathway genes, vernalization pathway genes, gibberellic acid pathway genes, and floral integrator genes, under short days (SD) for 28 d (from vegetative to reproductive transition stage); however, the expression of the autonomous pathway genes was not affected. This study provides the first evidence of a role for *ASR* genes in delayed flowering time in plants.

## Introduction

Flowering time is crucial for pollination and reproductive success in higher plants [1, 2], which is regulated through four major pathways, the photoperiod-, vernalization-, autonomous-, and gibberellic acid (GA)-dependent pathways, in *Arabidopsis* [3–5]. Photoperiod, or the duration of light in a given day, is a critical cue that flowering plants utilize to effectively assess seasonal information and coordinate their reproductive development in synchrony with the external environment [6]; photoperiod thus controls flowering time by regulating the expression of a number of key genes, such as *CONSTANS* (*CO*), *EARLY FLOWERING4* (*ELF4*), and *EARLY FLOWERING3* (*ELF3*) [6, 7]. Flowering in some plants can be stimulated by exposure to long periods of low non-freezing temperatures, which is known as varnalization, and is regulated by the *FRIGIDA* (*FRI*) and *FLOWERING LOCUS C* (*FLC*) genes [4, 8]. However, the autonomous pathway associated with the GA pathway integrates developmental signals to regulate plant flowering time [9]. Recent studies have reported that floral regulatory pathways regulate the expression of floral integrator genes such as *SUPPRESSOR OF OVEREXPRESSION OF CO1* (*SOC1*) and *LEAFY* (*LFY*) [10, 11]. In addition, a series of transcription factors including *CO*, *FLC*, *SQUAMOSA PROMOTER BINDING PROTEIN-LIKE* (*SPL*), and *CAPRICE* (*CPC*) control flowering time by regulating the target genes expression of these pathways [6, 10, 12–14].

The ABA-, stress-, and ripening-induced (ASR) protein is a plant-specific hydrophilic transcriptional factor widely distributed to approximately 20 monocot, dicot, and gymnosperm plant species belonging to the group 7 of the LATE EMBRYONGENESIS ABUNDANT proteins [15, 16]. It is a small size protein (~13 kDa) localized to both the nucleus and the cytoplasm, and contains Zn^2+^-dependent DNA binding activity at the N-terminus and a nuclear localization signal at the C-terminus [17, 18]. The number of *ASR* orthologous genes varies from 1 to 9 in different plant species [18] but, surprisingly, orthologs have not been identified in *Arabidopsis thaliana* and crucifer *Thlaspi caerulescens* [16]. Several *ASR* orthologous and paralogous genes are involved in fruit ripening and in the response to various abiotic stresses, particulary salt and drought stress tolerance [15–18]. Increasing evidence has also indicated that *ASRs* are involved in the regulation of floral development [19, 20]. In lily, ASR orthologous proteins accumulate only at the later stage of pollen maturation and these levels remain steady in mature and vital pollen [21]. Tomato *ASR1* and *ASR4* are expressed in flower organs [19], and tobacco ASR *in vivo* binds to a transcription factor bZIP involved in floral development [20]. Overexpression of the *ASR* gene affects sugar trafficking, flower development, and fruit development [18, 20, 22]; however, the role of *ASR* in regulating plant flowering time has not been reported.

Banana (*Musa* spp.), the second ranking fruit crop in the world, has a large, dark purple-red inflorescence and produces female, male, and bisexual flowers. Bud differentiation and fruit yield are largely determined by female flowering time. Our previous studies showed that *MaASR* enhances drought stress tolerance [16]. In the present study, we found that the overexpression of *MaASR* in *Arabidopsis* could result in a clear delayed-flowering phenotype. Microarray and quantitative real-time PCR (qRT-PCR) results demonstrated that the expression of a number of key genes involved in the flowering regulator pathways, including photoperiod-, vernalization-, GA-pathways, and floral integrator, are down-regulated by *MaASR* overexpression to delay flowering time. This study has identified the role of *ASR* genes in delayed flowering time for the first time, and this finding may enable regulation of flowering time in plant breeding and a genetic improvement of plant yields.

## Materials and Methods

### Plant materials

Banana (*M*. *acuminata* L. AAA group, cv. ‘Dwarf Cavendish’) (ITC0002) inflorescence was obtained from a banana plantation (Institute of Tropical Bioscience and Biotechnology, Chinese Academy of Tropical Agricultural Sciences, Haikou, Hainan province, China). Roots, leaves, rhizomes, fruits, and female flowers from the tenth (F10), ninth (F9), eighth (F8), fourth (F4), and first (F1) cluster of the inflorescence were collected to analyze *MaASR* expression. All materials were separately frozen in liquid N_2_ and stored at -80°C until later analysis.

*A*. *thaliana* (Columbia ecotype) seeds were purchased from the *Arabidopsis* Biological Resource Center (Ohio University, Columbus, USA). DH5α *Escherichia coli* and LBA4404 *Agrobacterium tumefaciens* strains were provided by Professor Jiaming Zhang from the Chinese Academy of Tropical Agricultural Sciences. All *Arabidopsis* seeds were sown on a 1:1:8 mixture (by weight) of vermiculite, perlite, and peat moss, respectively, and were grown at 22°C with 70% humidity and short day condition (SD, 8 h light/16 h dark cycle) illuminated by Sylvania GRO LUX fluorescent lamps (Utrecht, Netherlands). When *A*. *thaliana* produced 12–14 rosette leaves, they were grown at 70% humidity and long day (LD) condition with 16 h light/8 h dark cycle to promote flowering.

### Cloning, subcellular localization and expression analysis of *MaASR*

The full-length cDNA encoding *MaASR* was amplified with the primers MaASR-F and MaASR-R (S1 Table) based on the expressed sequence tag (EST) of *MaASR* isolated from a banana fruit cDNA library [23] with the adapter primers Ptr-F and Ptr-R (S1 Table). The *MaASR* full-length cDNA sequences were submitted to GenBank (http://www.ncbi.nlm.nih.gov/Banklt/index.html). Amino acid sequences were compared using the DNAMAN software package (Version 5.2.2, Canada).

The Open Reading Frame (ORF) of *MaASR* was inserted into a pCAMBIA1304-GFP expression vector to generate a MaASR-GFP fusion protein under the control of a cauliflower mosaic virus (CaMV) 35S promoter. The recombinant plasmid was transferred to the *A*. *tumefaciens* strain LBA4404 and introduced into *Nicotiana benthamiana* leaves as described previously by Goodin et al. [24]. After 48 h of incubation on MS at 25°C, fluorescence was examined using fluorescence microscopy (LSM700, Carl Zeiss, Germany).

*MaASR* expression was assayed by qRT-PCR in an iQ5 real-time PCR detection system (Bio-Rad, USA) using the SYBR ExScript RT-PCR kit (TaKaRa, Japan). A series of primer and template dilutions were performed to acquire the optimal primer and template concentrations for amplifying the target genes prior to quantification experiments. Primers that had high specificity and efficiency on the basis of melting curve analysis were used to conduct quantification analysis (S1 Table). Moreover, PCR products were sequenced to confirm the specificity of primer pairs. Amplification efficiencies of primer pairs ranged from 0.9 to 1.1. ACTIN (accession No. EF672732) and UBQ (accession No. XP009390884.1) that were verified to be constitutive expression and suitable to be used as internal controls were used as reference genes to normalize transcriptional levels of *MaASR* gene (S1 Table). The relative expression levels of *MaASR* gene were verified in triplicate and calculated using the 2^−ΔΔ*C*T^ method [25].

### Plant transformation and blot analysis of transgenic plants

A *MaASR* coding region driven by a 35S promoter was inserted into the pBI121 vector by replacing the β-glucuronidase following digestion with *Bam*HI and *Sac*I. The pBI121-MaASR was transferred into an *A*. *tumefaciens* strain LBA4404. Transgenic *Arabidopsis* plants were generated using the floral dip-mediated infiltration method [26]. Homozygous T_3_ kanamycin-resistant lines L14 and L38 were used for blot analyses and functional investigation.

Southern blot was used to determine the integration of *MaASR* to the *A*. *thaliana* genome. Probes were prepared from the PCR product using primers (5′-ccgaggagaagcaccaccac-3′ and 5′-gccaccgctgcagcgatctcc tc-3′) and were labelled with DIG-dUTP according to the manufacturer’s instructions (Roche Applied Science, Germany). A Northern blot probe was labelled using a random primer labeling system (Roche Applied Science, Germany) and hybridized according to the manufacturer’s instructions (Roche Applied Science, Germany). Western blotting was performed using MaASR monoclonal antibodies (Abmart, China) diluted 1:500. After hybridization, the membrane was washed and exposed to X-ray film (Kodak BioMax MS system) according to Miao et al. [27].

### Phenotype observation of *MaASR* transgenic plants

Rosette leaves number, bolting, and flowering time of *MaASR* transgenic lines L14 and L38 and WT were analyzed according to the methods of Diallo et al. [28]. The statistical analysis is listed in S2 Table. Vegetative growth, bolting, and flowering phenotype of the transgenic lines and WT were photographed. Floral organs phenotypic differences of early flowering between transgenic lines and WT under LD condition were observed using a dissecting microscope (OLMPUS-SZX12).

### Total RNA extraction and cDNA synthesis from *MaASR* transgenic lines and WT

RNAs were isolated from *MaASR* transgenic plants L14 and L38 and WT at 14 d and 28 d under the SD, 14 d and 28 d under the LD condition, respectively, using a plant RNA Kit (QIAGEN, Germany). The first strand of cDNA was synthesized using a RevertAidTM First Strand cDNA Synthesis Kit (Fermentas, Ontario) according to the manufacturer’s instructions. RNA quality was assessed by the fractionation of total RNA on a 1.2% (w/v) agarose gel and imaged using the GelDox XR system (Bio-Rad, USA). All RNA samples prepared (A260/A280 ratio = 1.8~2.0; rRNA ratio (28S/18S)>0.9) were suitable for microarray and expression analysis.

### Microarray profiling and data analysis

cDNAs prepared from *MaASR* transgenic lines L14 and WT were used for microarray analysis. Each sample included three biological replicates for L14 (designed as L14-1, L14-2, and L14-3) and WT (designed as WT1, WT2, and WT3). Labeling and hybridization was performed using the 29 k *Arabidopsis* Genome Array (Arabidopsis thaliana Genome Oligo Set Version3.0, http://www.operon.com) according to the procedure described by Patterson et al. [29]. Controls were also printed on glass slides using a SmartArray microarrayer (CapitalBio Corp.). The resulting images were analyzed with LuxScanTM3.0 software (CapitalBio Corp.) and identified using the methods of Miao et al. [30]. DEGs were identified using a *P* value <0.05, false discovery rate (FDR) <0.05, and a fold change ≥2.0.

### qRT-PCR analysis of flowering-related pathway genes

Primers that had high specificity and efficiency on the basis of melting curve analysis and agarose gel electrophoresis were designed with Primer premier 5.0 software (http://www.premierbiosoft.com/) and used to conduct quantification analysis (S1 Fig). Primer sequences of *AtFCA*, *AtFLK*, *AtFRI*, *AtGAI*, *AtLFY*, *AtRGL1*, *AtVRN1*, *AtFLC*, *AtFVE*, *AtSOC1*, *AtCol1*, *AtCol2*, *AtNAP*, *AtTCH2*, *AtSEP3*, *AtCol9*, *AtCO*, *AtELF3*, *AtELF4*, *AtNGA1*, and *AtMAF5* are listed in S1 Table. Amplification efficiencies of primer pairs ranged from 0.9 to 1.1. The *AtACTIN* and *AtUBQ* [31] that were verified to be constitutively expressed and suitable for use as internal controls were as reference genes to normalize transcriptional levels of target genes in this study (S1 Table). The relative expression of the tested genes with three replicates of each sample was assessed according to the 2^−ΔΔ*C*T^ method [25].

### Statistical analysis

For all generated data, at least three biological replicates were performed for each sample. Then, one-way ANOVA and Duncan’s multiple range tests were performed at a 5% significance level (*P* values <0.05) using SPSS software (version 13.0). The statistical results were reported as mean±SD.

## Results

### Sequence analysis, subcellular localization and expression pattern of *MaASR* in banana female flowers

The cDNA of *ASR* from banana, *MaASR*, was 747 bp in length containing a 432 bp open reading frame (ORF), which encoded a protein of 143 amino acids with a 251 bp 5′ untranslated region (UTR) and a 64 bp 3′ UTR. The sequences of the cloned *MaASR* were registered in GenBank under the accession No. AY628102. Compared to the other ASR amino acid sequences, *MaASR* contained a small N-terminal DNA binding site (HHHRLFHH) and a longer putative nuclear C-terminal localization signal (KRDAKNEAEEASGKKHHHHL) (S2 Fig). MaASR protein was localized in the nucleus and plasma membrane (S3 Fig).

**Fig 1:**
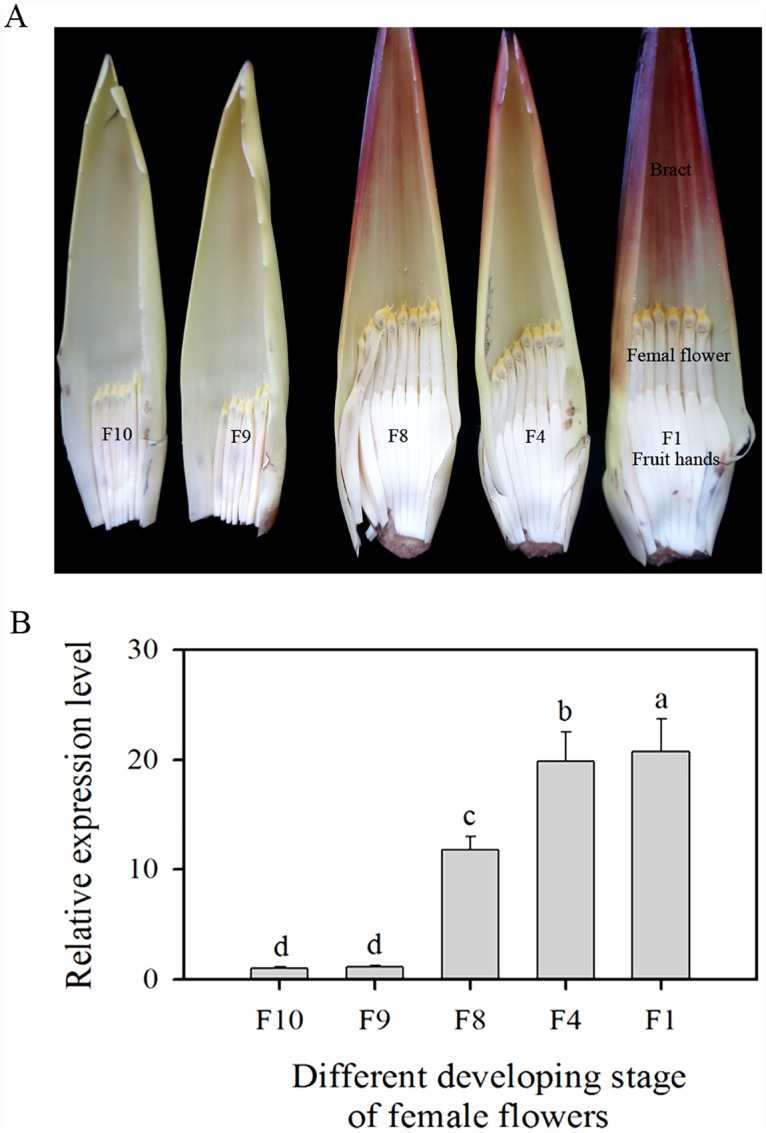
Expression of *MaASR* gene in banana female flowers from the upper inflorescence. (A) The female flowers from the tenth (F10), ninth (F9), eighth (F8), fourth (F4), and first (F1) cluster from the upper inflorescence. (B) Relative expression level in banana female flowers. The y-axis represents the relative fold-difference in mRNA level, which was calculated using the 2^-ΔΔCT^ formula with ACTIN and UBQ as internal controls. The vertical bars represent the mean ± SD of three replicates.

*MaASR* expression was detected in roots, leaves, rhizomes, female flowers (the first cluster from the upper inflorescence), and fruits. Female flowers showed the highest levels, along with roots; the lowest level was found in rhizomes. The level of *MaASR* expression in flowers was approximately 12-fold higher than that in rhizomes (S4 Fig). Significant differences in *MaASR* expression were detected in the female flowers from the tenth (F10), ninth (F9), eighth (F8), fourth (F4), and first (F1) cluster from the upper inflorescence (Fig 1A). At F10, the *MaASR* gene began to express and reached a maximum at F1, which was approximately 20-fold higher than that at F10 and F9 (Fig 1B), suggesting that expression of *MaASR* could play a role in banana female flower development.

### *MaASR* overexpression caused a clear delayed flowering phenotype

**Fig 2:**
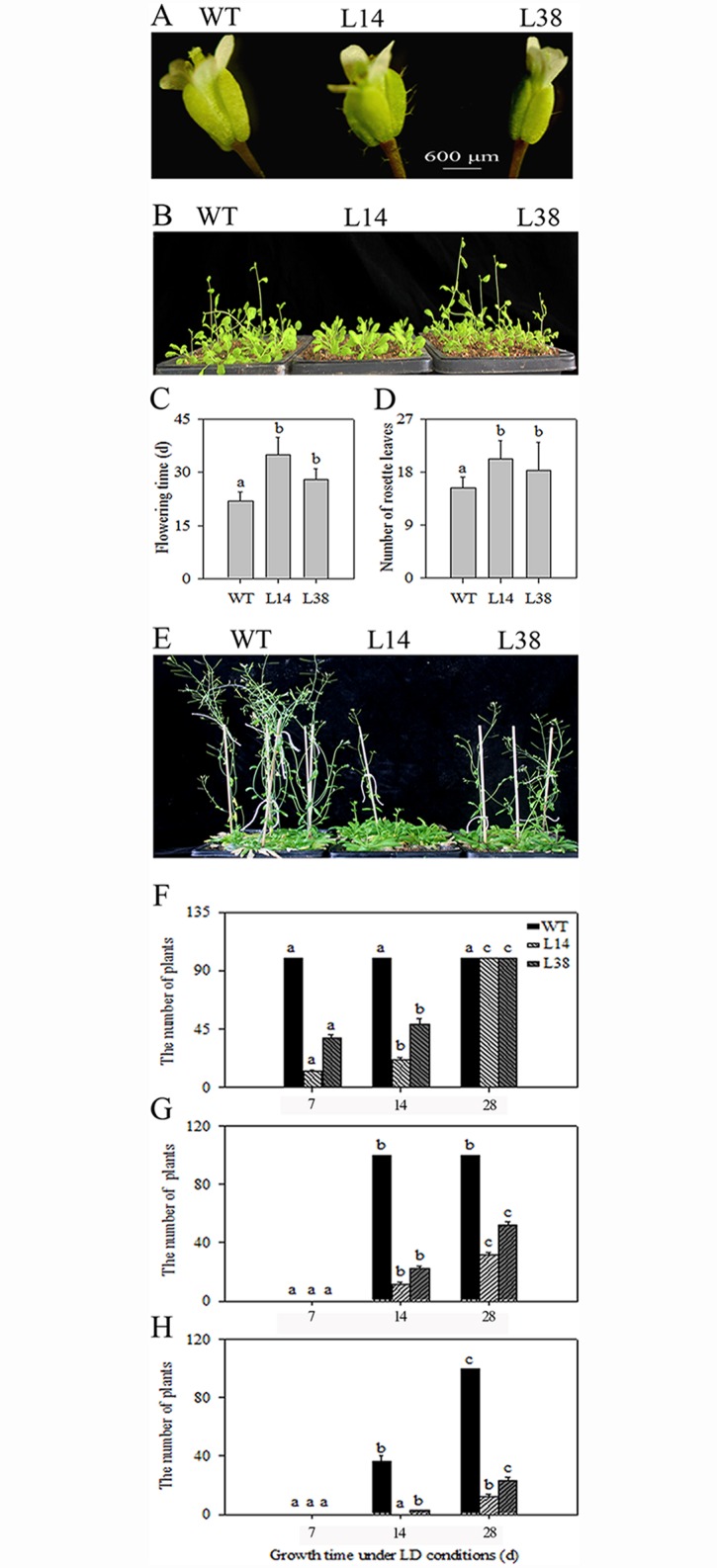
Flowering phenotypes of *MaASR* transgenic plants. (A) Phenotype of floral organs detached from the same position and developmental stage of 14 d under LD in WT and transgenic plants. (B) Plants at 14 d under LD. (C) Flowering days of *MaASR* transgenic lines L14 and L38. (D) The number of rosette leaves of *MaASR* transgenic lines L14 and L38. (E) Plants at 28 d under LD. (F) The number of plants at flower buds stage. (G) The number of plants at mid-flower stage. (H) The number of plants at full-bloom stage.

Floral organs of two transgenic lines, L14 and L38, were significantly shorter (0.67-fold) than that of the WT (Fig 2A). The number of sepal and pedicel trichomes in the L14 line was greater than that of the L38 line. These trichomes are rarely present in the sepal and pedicel of WT, indicating that floral organ morphological changes in transgenic lines are relavant to the overexpression of *MaASR*. Southern blot showed that L14 and L38 harbored two and one copies of *MaASR*, respectively (S5 Fig). Northern and Western blots confirmed that MaASR transcripts were present in the two transgenic lines compared to WT in which expression was absent (S5 Fig).

Under 7 d of LD conditions, most WT plants, as well as the *MaASR* transgenic line L38, displayed bolting but the L14 line did not (Fig 2B). The number of days required for bolting was 22, 35, and 28 d in WT, L14, and L38, respectively (Fig 2C). The average number of rosette leaves in the WT, L14, and L38 lines was 15, 20, and 18, respectively (Fig 2D). These results showed that the number of rosette leaves produced by the *MaASR* transgenic lines was significantly greater than those produced by WT.

Under 28 d of LD conditions, WT reached the full-bloom stage and pods were observed; however, the bolting number of the L14 and L38 lines was significantly lower than that of WT (Fig 2E). Statistical analyses showed that flower buds were formed in WT under LD conditions for 7 d. The L14 and L38 lines only formed 12.76% and 38.33% of flower buds, respectively, until 28 d, at which point the flower buds of the transgenic lines were fully formed (Fig 2F). Under LD conditions for 14 d, all flower buds from WT were at mid-flower stage; the number of flower buds at the mid-flower stage in lines L14 and L38 was only 11.17% and 22.24%, respectively (Fig 2G). By 28 d, WT reached full bloom, but the flower number in lines L14 and L38 was only 12.21% and 23.50%, respectively (Fig 2H). These data demonstrate that *MaASR* transgenic lines have a significantly delayed flowering phenotype with respect to flower buds at mid-flower and full bloom stages.

### Microarray analysis and screening of the differential expressed genes (DEGs) between *MaASR* transgenic plants and WT

**Fig 3:**
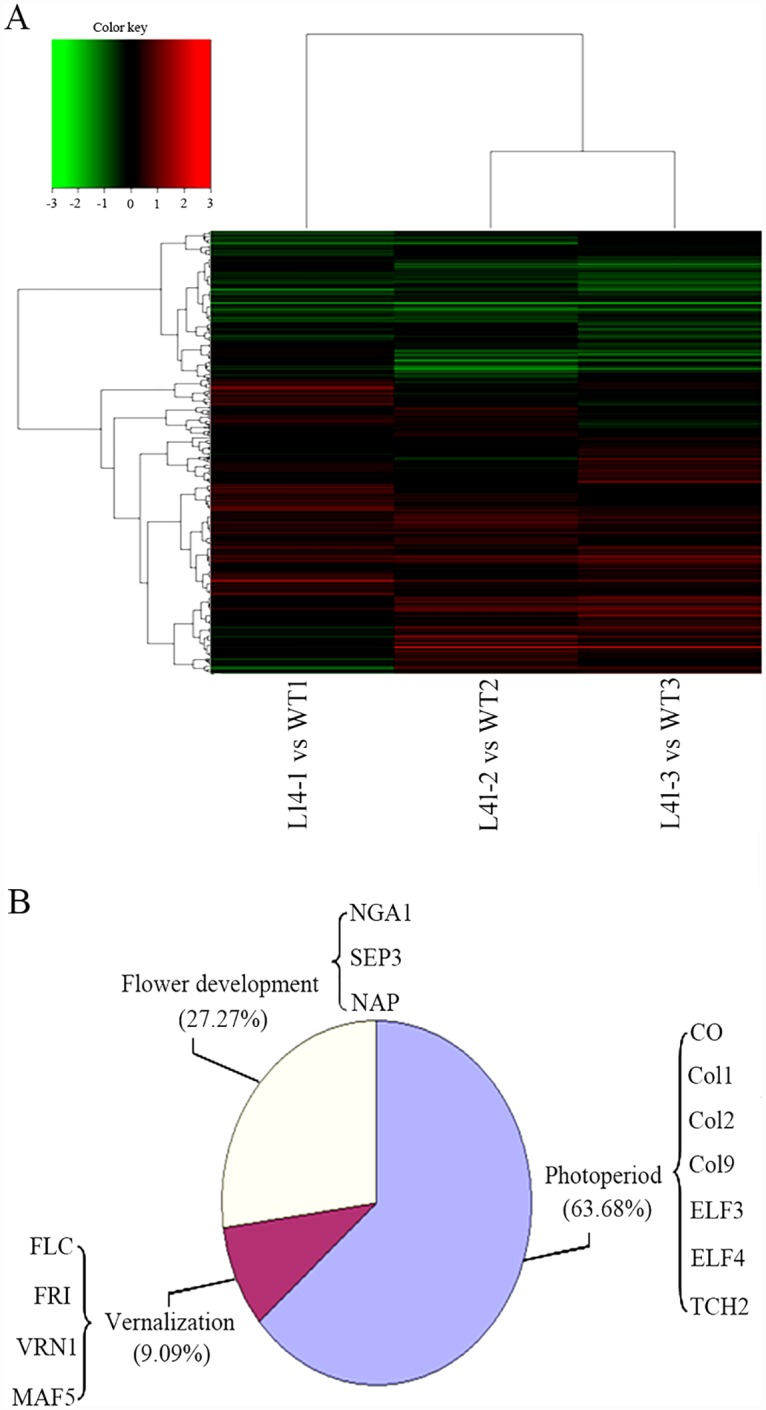
TreeView representation of ESTs from microarray data (L14 vs WT) and functional classification of flowering-related candidate genes. (A) Gene expression profile of transgenic plants L14 and WT. (B) Functional classification of candidate genes. Red: up-regulated genes; Green: down-regulated genes.

Microarray analysis was used to determine the DEGs affected by *MaASR* overexpression compared to the WT. Each sample included three biological replicates for L14 (designed as L14-1, L14-2, and L14-3) and WT (designed as WT1, WT2, and WT3). TreeView representation of L14-1-vs-WT1, L14-2-vs-WT2, and L14-1-vs-WT3 libraries is shown in Fig 3A. Based on a fold change ≥2.0 and *P* value < 0.05, a total of 747 DEGs were identified, including 559 up-regulated genes (S3 Table) and 188 down-regulated (S4 Table) genes in L14 vs WT. All DEGs were mapped to the Gene Ontology (GO) database with respect to biological processes, molecular functions, and cellular components (S6 Fig).

The 11 candidate genes involved in flowering included 6 up-regulated (*Col9*, *ELF3*, *ELF4*, *TCH2*, *NGA1*, and *NAP*) genes and 5 down-regulated (*CO*, *Col1*, *Col2*, *MAF5*, and *SEP3*) genes in L14 (Fig 3B). The 11 candidate genes were then divided into three pathways, the photoperiod pathways (*CO*, *Col1*, *Col2*, *Col9*, *ELF3*, *ELF4*, and *TCH2*), vernalization pathways (*MAF5*), and flowering development pathways (*NGA1*, *SEP3*, and *NAP*) (Fig 3B). These key pathway genes were subjected to further detailed expression analysis using qRT-PCR.

### Overexpression of *MaASR* reduces the expression of photoperiod pathway genes under SD for 28 d

**Fig 4:**
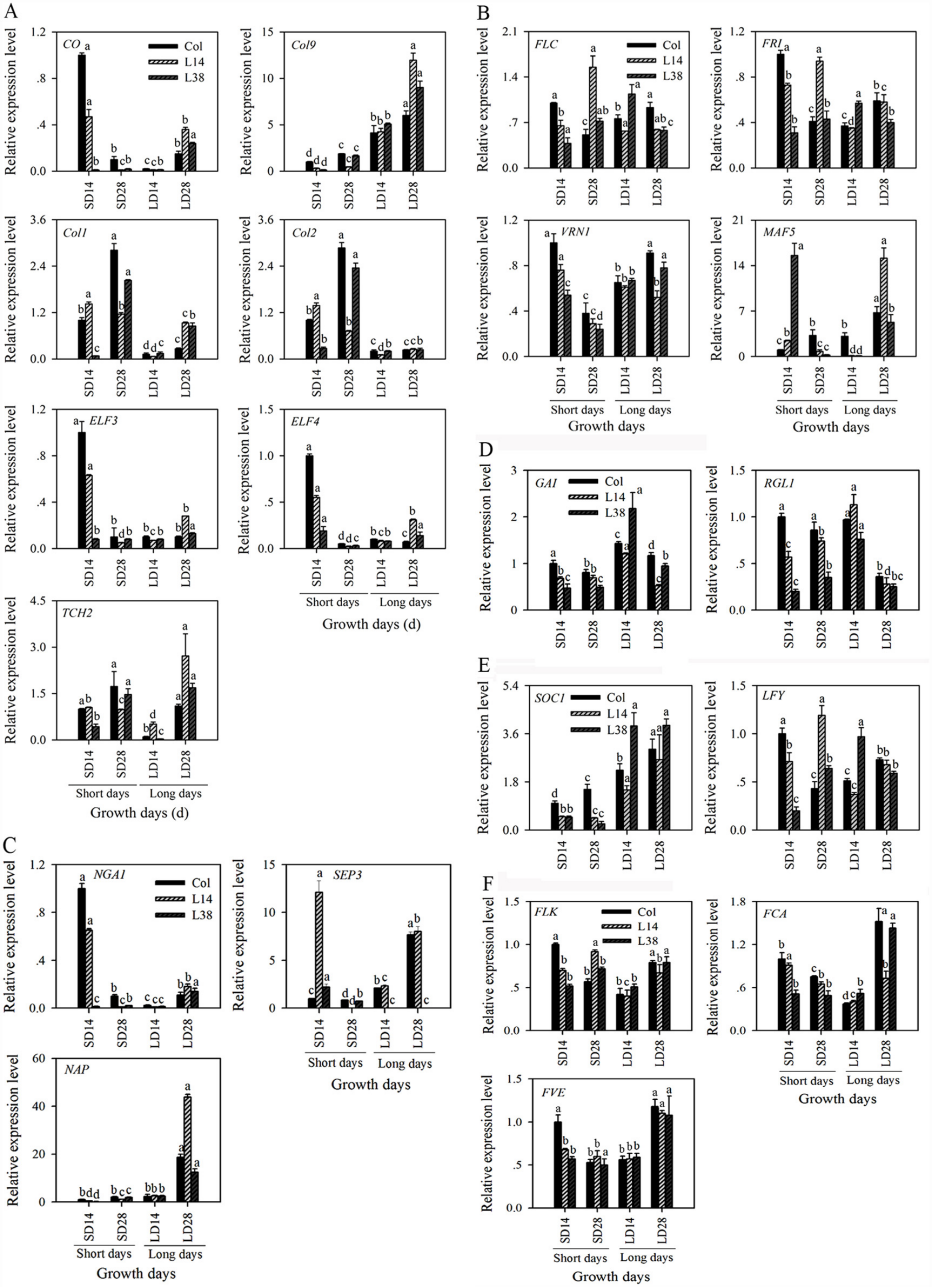
Expression analysis of photoperiod pathway genes, vernalization pathway genes, flower development related genes, GA pathway genes, floral integrator genes, and autonomous pathway genes in WT and *MaASR* transgenic plants. (A) Photoperiod pathway genes. (B) Vernalization pathway genes. (C) Flower development related genes. (D) GA pathway genes. (E) Floral integrator genes. (F) Autonomous pathway genes. WT: Wild-type; L14, L38: *MaASR* transgenic lines. Data are represented as mean ± SD of biological replicates (n = 3). Means denoted by the same letter do not significantly differ when set at *P*<0.05 as determined by Duncan’s multiple range tests.

The expression of seven photoperiod pathway genes (*CO*, *Col1*, *Col2*, *Col9*, *ELF3*, *ELF4*, and *TCH2*) was examined between WT and the *MaASR-*overexpressing transgenic plants (L14 and L38) (Fig 4A). The expression pattern of *CO* in WT and *MaASR* transgenic lines was similar to *Col1*, *Col2*, *ELF3*, *ELF4*, and *TCH2* but it was reversed with *Col9* (Fig 4A). Compared to WT, the expression levels of *CO*, *Col1*, *Col2*, *Col9*, *ELF3*, *ELF4*, and *TCH2* in *MaASR* transgenic lines were significantly lower than that of the WT under SD for 28 d (from vegetative to reproductive transition stage), specifically in the L14 line compared to the L38 line (Fig 4A), suggesting that different copy numbers of L14 and L38 can affect expression levels in transgenic plants. These results showed that overexpression of *MaASR* reduced the photoperiod pathway genes expression levels at 28 d under SD.

### Overexpression of *MaASR* reduces the expression of vernalization pathway genes (*VRN1*and *MAF5*) under SD for 28 d

Four key genes involved in vernalization pathway (*FLC*, *FRI*, *VRN1*, and *MAF5*) were screened by microarra**y** analysis based on previous studies of *Arabidopsis* [4, 32, 33]. *FLC* and *FRI* act as inhibitors of flowering in the vernalization pathway [4]. *VRN1* and *MAF5* could play an opposite role of *FLC* [32]. In Fig 4B, the expression of *FLC* and *FRI* was lower in the *MaASR* transgenic lines under SD for 14 d than that in WT. Transgenic plants exhibited enhanced expression of *FLC* and *FRI* compared to WT under SD for 28 d but the expression of *VRN1* and *MAF5* was lower in the transgenic lines under SD for 28 d, suggesting that *MaASR* overexpression increases *FLC* and *FRI* expression and decreases *VRN1* and *MAF5* expression under SD for 28 d to delay flowering time.

### Overexpression of *MaASR* alters the expression pattern of flower development related genes

Three flowering development pathway genes (*NGA1*, *SEP3*, and *NAP*) were screened by microarray analysis (Fig 3B). The expression levels of *NGA1*, *SEP3*, and *NAP* were lower in the transgenic lines under SD for 28 d compared to WT (Fig 4C). *SEP3* expression was significantly different between the WT and transgenic lines. *SEP3* expression gradually increased in the WT from 14 d under SD to 28 d under LD but its expression in the *MaASR* transgenic lines declined rapidly from 14 d under SD to 28 d under SD and then increased gradually in L14 from 14 d under LD to 28 d under LD (Fig 4C). These results suggest that *MaASR* overexpression suppresses the expression of flowering development pathway genes (*NGA1* and *NAP*), altering the expression pattern of *SEP3*.

### Overexpression of *MaASR* reduces expression of GA pathway genes and floral integrator genes under SD for 28 d, but did not affect expression of autonomous pathway genes

Based on previous studies in *Arabidopsis*, several GA pathway genes (*GAI* and *RGL1*), floral integrator genes (*SOC1* and *LFY*), and autonomous pathway genes (*FLK*, *FCA*, and *FVE*) have been identified by qRT-PCR [34, 35]. The expression levels of *GAI* and *RGL1* in transgenic plants at 14 d under SD, 28 d under SD, and 28 d under LD were lower than in the WT (Fig 4D). *SOC1* expression was lower in transgenic lines before flowering (from 14 d under SD to 28 d under SD) compared to WT; however, *LFY* expression was higher in the transgenic lines at 28 d under SD compared to WT (Fig 4E). *FLK*, *FCA*, and *FVE* promote flowering via negative regulation of *FLC* transcriptional levels in the autonomous pathway [36]. *FLK*, *FCA*, and *FVE* expression revealed similar trends from 14 d under SD to 28 d under LD between WT and transgenic plants (Fig 4F), indicating that *MaASR* overexpression reduces the expression of GA pathway genes (*GAI* and *RGL1*) and floral integrator gene (*SOC1*) at 28 d under SD but does not affect the expression of autonomous pathway genes.

### A tentative model of the main genes involved in the flowering pathway in *MaASR*-overexpressed plants

**Fig 5:**
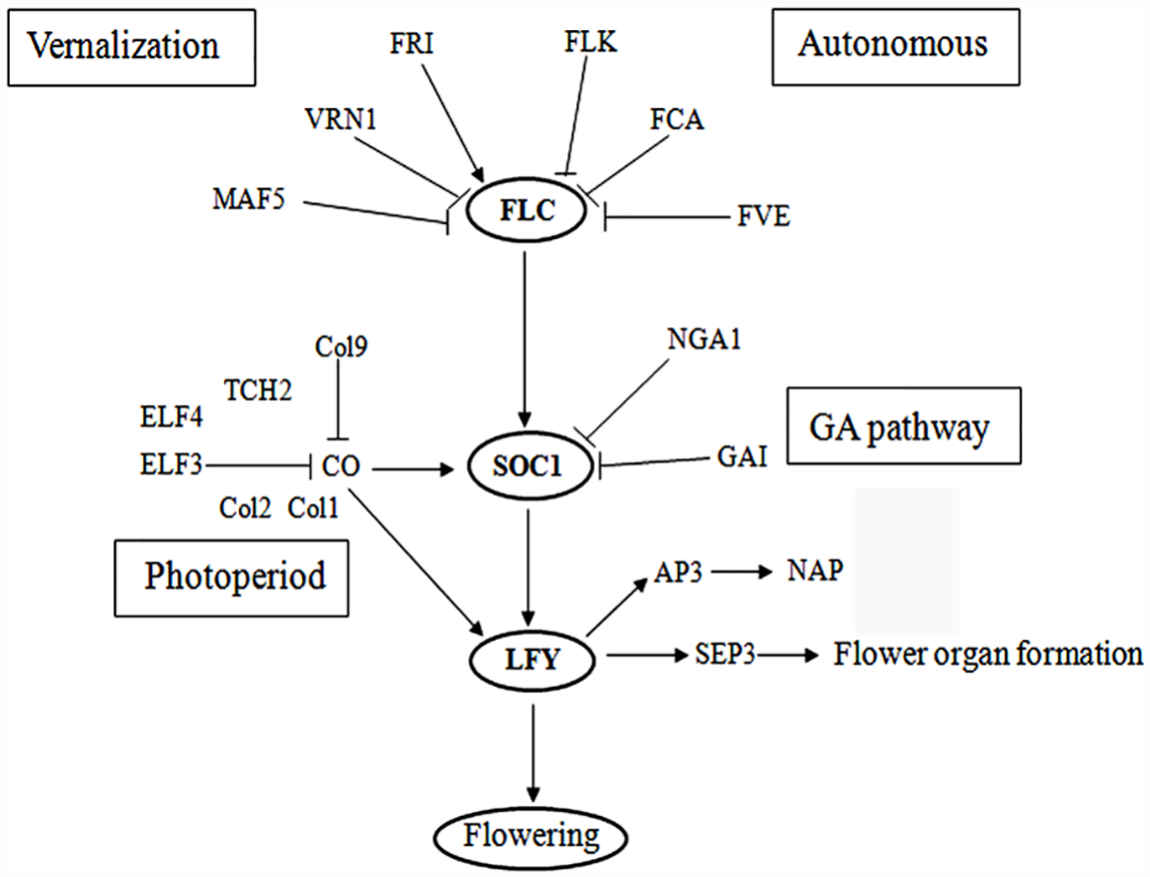
A tentative model showing the main genes involved in the multiple flowering pathway in *MaASR* overexpressed plants.

A tentative model of the flowering regulatory network associated with *MaASR* overexpression was developed (Fig 5). Three genes, *FLC*, *SOC1* and *LFY*, are in the core of the network. In the model, photoperiod-related genes (*CO*, *ELF3*, *ELF4*, *Col1*, *Col2*, *Col9*, and *TCH2*) inhibit flowering time by repressing *SOC1* and *LFY* expression in *MaASR* transgenic plants at 28 d under SD condition. Vernalization pathways primarily access the network by inhibiting expression of *FLC*. GA pathway genes inhibit the expression of *SOC1*. Flower developmental pathway genes are directly regulated by *LFY* to affect flower organ formation.

Autonomous pathway genes regulate flowering time by affecting *FLC* expression between WT and transgenic plants but *MaASR* overexpression does not affect expression of autonomous pathway genes. These findings indicate that *MaASR* delays flowering time by reducing expression of several photoperiod pathway genes, vernalization pathway genes, and GA pathway genes, while expression of several other flower development related genes (*NGA1*, *SEP3*, and *NAP*) and floral integrator genes (*SOC1*) are inhibited by *MaASR* overexpression.

## Discussion

Despite extensive studies on the role of *MaASR* in fruit-ripening and in response to various abiotic stresses (mainly salt and drought stress tolerance) [16, 23], prior to this study, *MaASR*’s role in regulating flowering time in bananas was not studied. For the first time, herein we demonstrated that *MaASR* overexpression resulted in a clear delayed-flowering phenotype. The numbers of rosette leaves, sepal, and pedicel trichomes in transgenic *Arabidopsis* plants, L14 and L38, were significantly greater than those of WT under LD conditions. Similar observations have been made for other key flowering genes, such as *FLC* and *AERIAL ROSETTE I* (*ART1*), in causing enlarged basal rosette of leaves, developed adaxial trichome formation, and floral reversion for delayed flowering [37, 38]. Additional results showed that delaying in flowering time due to *MaASR* overexpression was caused mainly by the attenuated expression of several photoperiod pathway genes (*CO*, *Col1*, *Col2*, *ELF3*, and *ELF4*), vernalization pathway genes (*VRN1* and *MAF5*), flowering development pathway genes (*NGA1*, *SEP3*, and *NAP*), GA pathway genes (*GAI* and *RGL1*), and floral integrator genes (*SOC1*) under SD for 28 d (from vegetative to reproductive transition stage). Interestingly, the expression of autonomous pathway genes was not affected.

*CO* is an important floral regulator in the photoperiod pathway, integrating the circadian and light signals to control flowering time in the early stage of *Arabidopsis* growth [39, 40]. *Col9*, *Col1*, and *Col2* encode zinc finger proteins and are homologous genes of *CO*; *Col9* delays flowering by reducing *CO* expression in *Arabidopsis* and over-expression of *Col1* and *Col2* can shorten the period of circadian rhythms [40, 41]. *ELF3* and *ELF4* negatively regulate *CO* transcription [42, 43]. In this study, *Col9* expression showed a reverse pattern with *CO* expression, as in *Arabidopsis* [40]. While the expression levels of *CO*, *Col1*, *Col2*, *ELF3*, and *ELF4* in *MaASR* transgenic plants were significantly lower than that of the WT under SD for 28 d (from vegetative to reproductive transition stage) (Fig 4A), overexpression of *MaASR* reduced several photoperiod pathway gene expression to prevent the switch from vegetative to reproductive growth, consequently delaying flowering time; this result was also supported by microarray analysis (Fig 3), indicating that photoperiod pathway may play a pivotal role in the regulating flowering time of *MaASR*. Further experiments will be required to determine the interaction mechanism between photoperiod pathway genes and *MaASR*.

*FLC* is an inhibitor of flowering in the vernalization pathway by binding the *SOC1* promoter to regulate flowering time in *Arabidopsis* [4]. The *FRI* increases *FLC* levels and affects flowering time [4]. *VRN1* is responsive to low temperature and could participate in the vernalization pathway to help regulate flowering time [33]. *MAF5* could play an opposite role to *FLC* in the vernalization response [32]. In this study, the expression levels of *FLC* and *FRI* were higher in *MaASR* transgenic lines than in the WT at 28 d under SD conditions but *VRN1* and *MAF5* expression levels were lower in transgenic lines than in the WT at 28 d under SD conditions (Fig 4B), suggesting that *MaASR* overexpression could increase *FLC* and *FRI* transcription and reduce *VRN1* and *MAF5* expression levels at 28 d under SD conditions to delay flowering.

*NGA1* belongs to the AP2 transcription factor family and inhibits stigma and style development via negative regulation of *SOC1* expression in *Arabidopsis* [44]. In this study, the expression levels of *NGA1* in *MaASR* transgenic lines were lower from 14 d to 28 d under SD compared to WT (Fig 4C), suggesting that *MaASR* overexpression may affect floral development by repressing *NGA1* expression. *SEP3* affects floral organ formation by controlling *LFY* expression [45]. Here, the expression pattern of *SEP3* was significantly different between WT and the transgenic lines. *SEP3* expression gradually increased in WT, but its expression in the transgenic lines declined rapidly from 14 under SD to 14 under LD (Fig 4C), suggesting that *MaASR* overexpression altered expression pattern of *SEP3* to affect floral organ formation. Further studies are required in order to fully understand the interaction between the regulatory networks in *MaASR* overexpression and other flowering development-related pathway genes.

*GAI* and *RGL1* belong to the DELLA subfamily and are negative regulators of GA in the flowering process [34]. Here, the expression levels of *GAI* and *RGL1* in *MaASR* transgenic plants were found to be lower than that in WT at 28 d under SD conditions (Fig 4D), as in *Arabidopsis* [34]. *SOC1*, encodes a MADS box transcription factor and integrates multiple flowering signals derived from photoperiod, temperature, and hormone signals to prevent premature differentiation of the floral meristem [35]. In this study, *SOC1* expression was lower in *MaASR* transgenic lines before flowering (from 14 d under SD to 28 d under SD) compared to the WT (Fig 4E). *LFY* is a master regulator of flowering and of flower development, and acts as part of a switch that mediates the transition from the vegetative to the reproductive phase of plant development [46]. Here, *LFY* expression levels were lower in *MaASR* transgenic lines under SD for 14 d before the transition from the vegetative to the reproductive stage (Fig 4E), suggesting early *MaASR* overexpression was repressed from the vegetative to the reproductive phase transition by reduced *LFY* expression.

*FLK*, *FCA*, and *FVE* are three members of an autonomous pathway that cause a late-flowering phenotype in *Arabidopsis* [36]; however, some mutations in *FLK* gave rise to phenotypes with only slightly delayed flowering [47]. *FCA* interacts with *FY* in regulating flowering time [48] and *FVE* participates in the regulation of flowering time by repressing *FLC* transcription [49]. In this study, the expression patterns of *FLK*, *FCA*, and *FVE* genes were similar between *MaASR* transgenic lines and WT (Fig 4F), suggesting that delayed flowering due to *MaASR* overexpression may not affect the expression of autonomous pathway genes.

## Conclusions

*MaASR* gene is isolated and characterized from banana. Subcellular localization analysis showed that MaASR protein was localized in the nucleus and plasma membrane. Differences in the expression of *MaASR* gene were detected in different developmental stages of banana female flowers. *MaASR* transgenic lines showed a clear delayed-flowering phenotype. Overexpression of *MaASR* was able to delay flowering time by reducing the expression of several genes, including photoperiod pathway genes, vernalization pathway genes, GA pathway genes, and floral integrator genes, under SD for 28 d during the transition period from vegetative to reproductive phase, but without affecting the expression of autonomous pathway genes. This study provides a new insight into the regulatory mechanisms of flowering time and warrants further studies on *MaASR* that may lead to the development of strategies to regulate flowering time in banana and other flowering plants.

## Supporting Information

S1 Fig PCR screening of qRT-PCR primers. (DOC)

S2 Fig Alignment of amino acid sequences of *ASR* genes from different plants. (A) Domain: Zn^2+^-dependent DNA binding site in the N-terminal. (B) Domain: a conserved nuclear localization signal in the C-terminal.
(TIF)

S3 Fig Subcellular localization of the MaASR fused with GFP. (A) Green fluorescence in dark field. (B) Green fluorescence in bright field.
(TIF)

S4 Fig *MaASR* expression levels in different tissues. (TIFF)

S5 Fig Blot analysis of *MaASR* overexpression plants. (A) Southern blot analysis of *MaASR* transgenic lines L14 and L38. (B) Northern blot analysis of *MaASR* expression in transgenic lines L14 and L38. (C) Western blot analysis of MaASR expression in transgenic lines L14 and L38.
(TIFF)

S6 Fig GO enrichment analysis of all DEGs in *MaASR* transgenic plants L14 compared to WT. (A) Cellular components. (B) Molecular functions. (C) Biological processes.
(TIFF)

S1 Table Primers used in the study. (DOC)

S2 Table Statistical plant numbers listed in this study. (DOC)

S3 Table Up-regulated genes in the expression profile microarray (L14 vs WT). (XLS)

S4 Table Down-regulated genes in the expression profile microarray (L14 vs WT). (XLS)
