# Supplementary figures and images for: A Novel Role for Banana MaASR in the Regulation of Flowering Time in Transgenic Arabidopsis
Source: PLoS One. 2016 Aug 3;11(8):e0160690. doi: 10.1371/journal.pone.0160690 (PMC4972433; doi:10.1371/journal.pone.0160690)

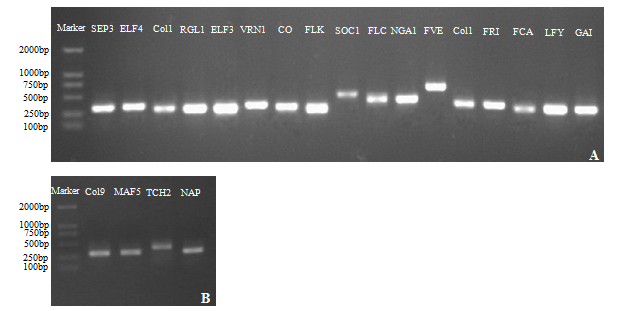


**Fig.S1**

Supplement: S1 Fig — (DOC) [file pone.0160690.s001.doc]

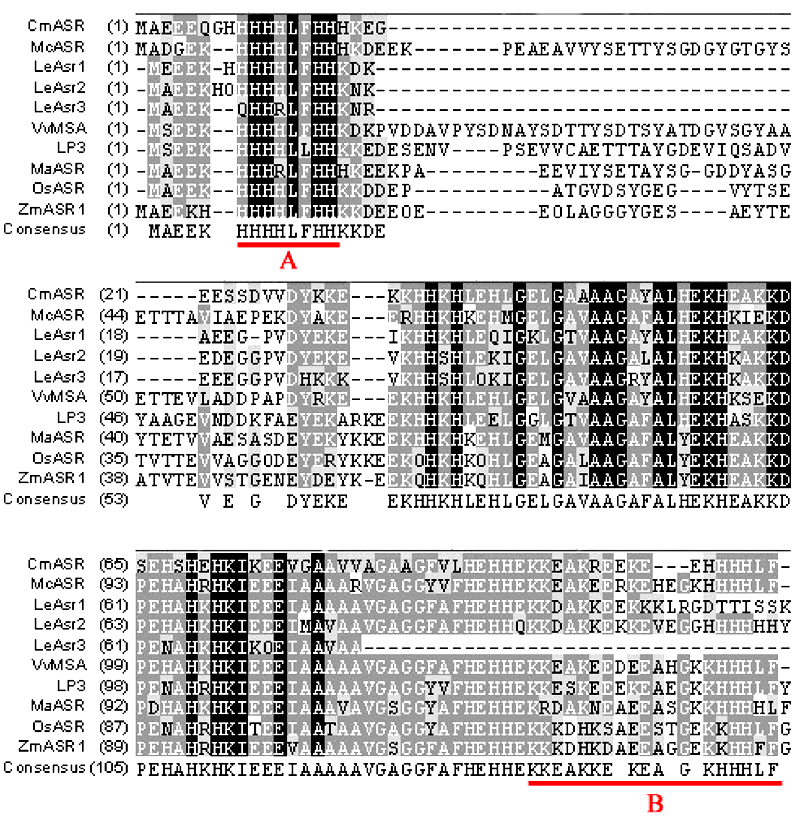

Supplement: S2 Fig — (A) Domain: Zn2+-dependent DNA binding site in the N-terminal. (B) Domain: a conserved nuclear localization signal in the C-terminal. (TIF) [file pone.0160690.s002.tif]

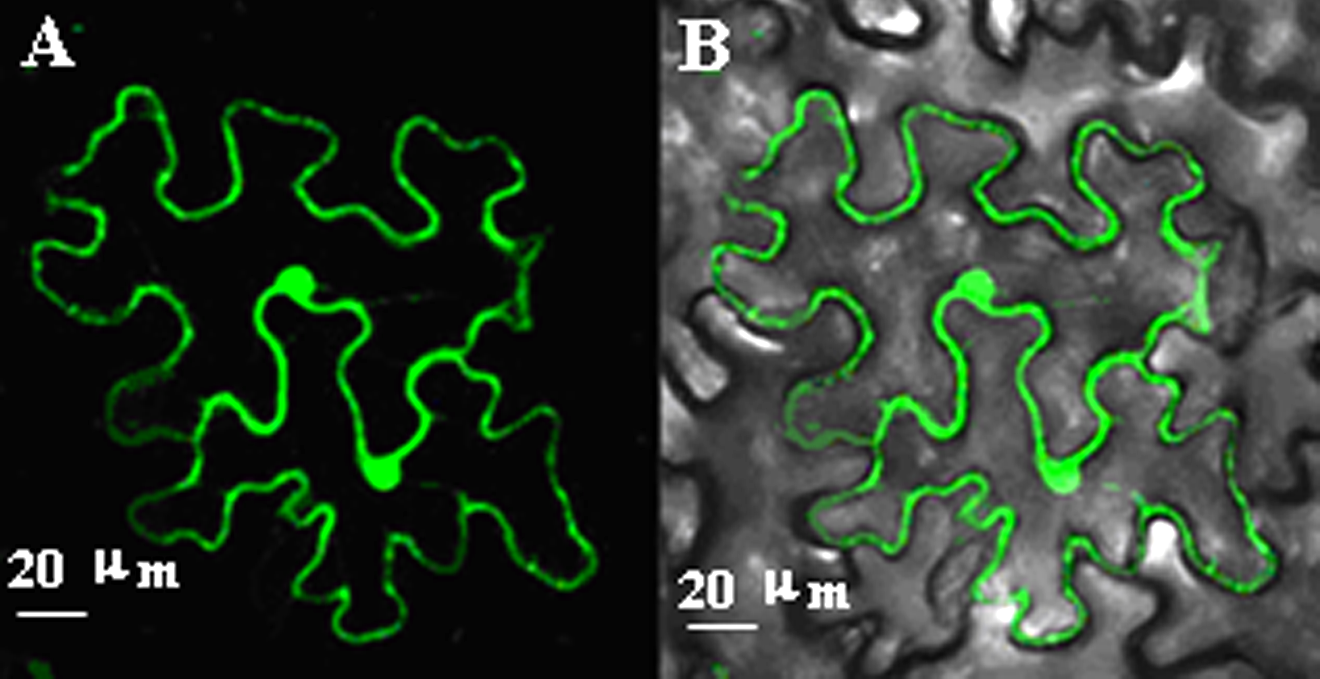

Supplement: S3 Fig — (A) Green fluorescence in dark field. (B) Green fluorescence in bright field. (TIF) [file pone.0160690.s003.tif]

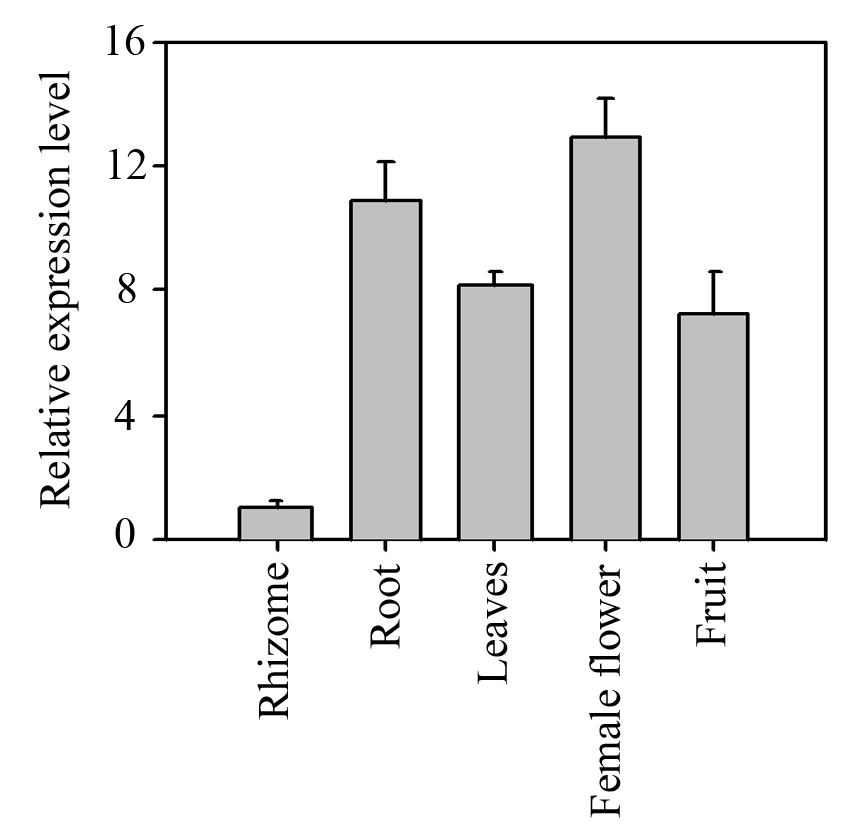

Supplement: S4 Fig — (TIFF) [file pone.0160690.s004.tiff]

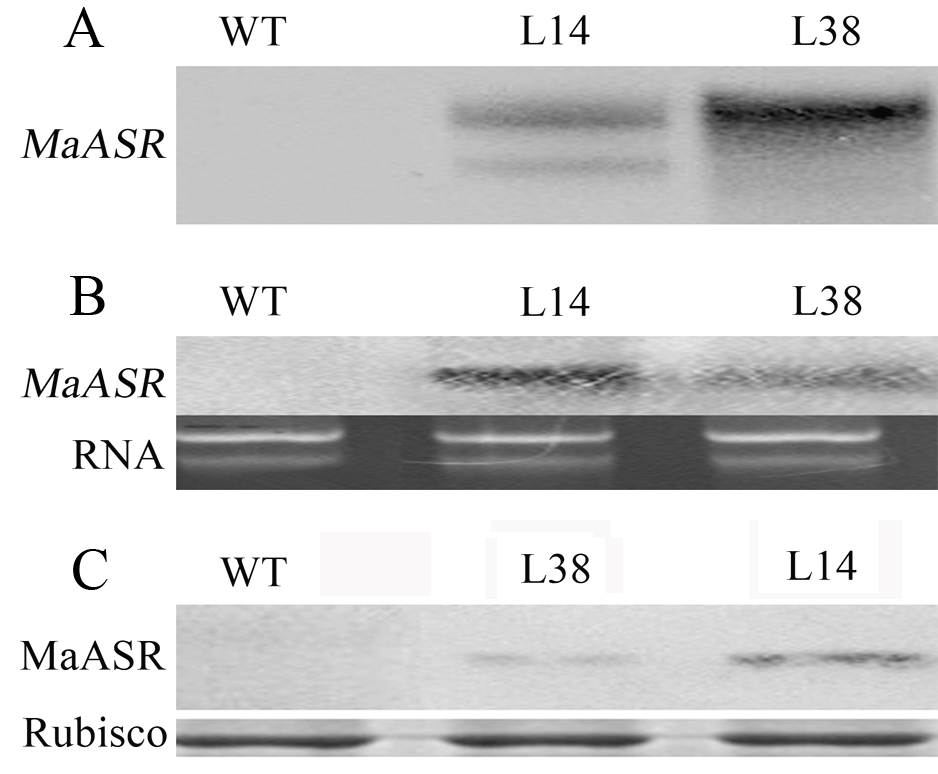

Supplement: S5 Fig — (A) Southern blot analysis of MaASR transgenic lines L14 and L38. (B) Northern blot analysis of MaASR expression in transgenic lines L14 and L38. (C) Western blot analysis of MaASR expression in transgenic lines L14 and L38. (TIFF) [file pone.0160690.s005.tiff]

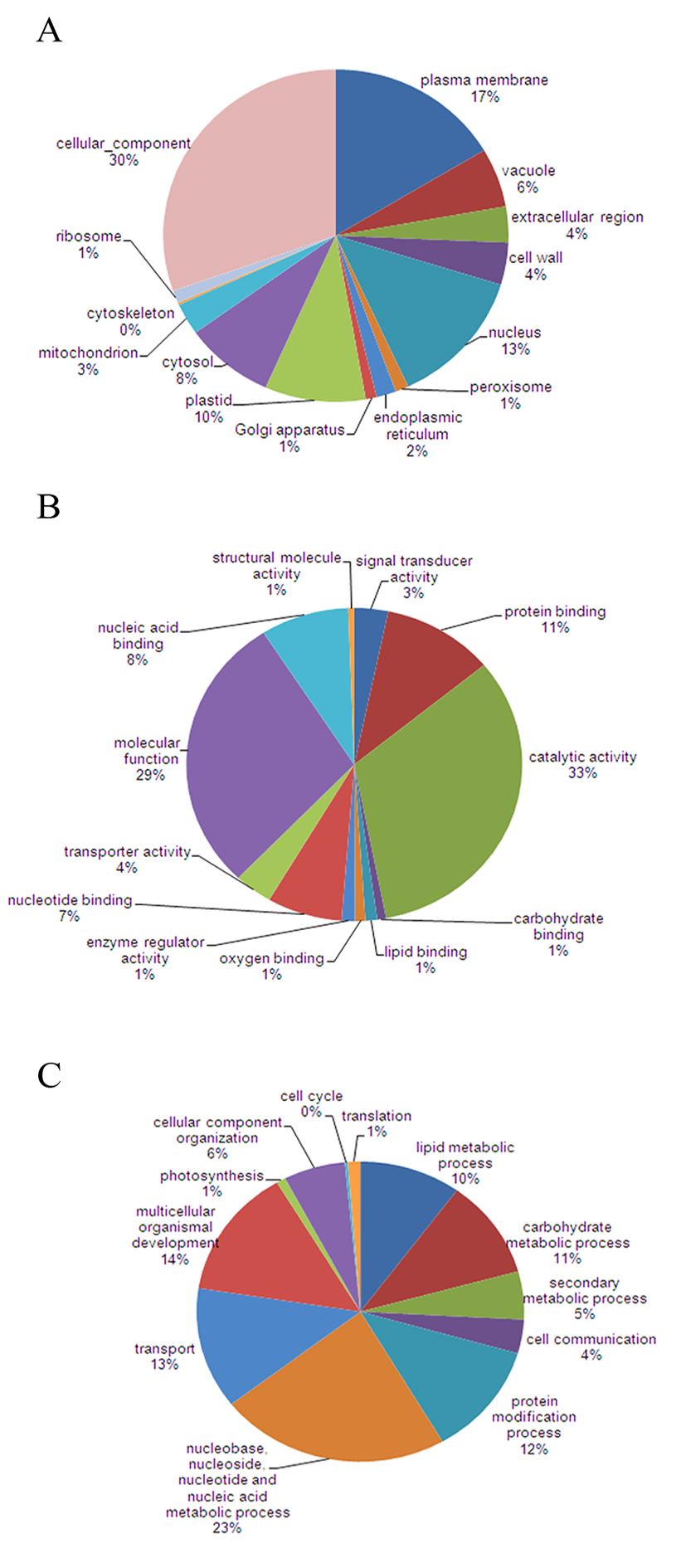

Supplement: S6 Fig — (A) Cellular components. (B) Molecular functions. (C) Biological processes. (TIFF) [file pone.0160690.s006.tiff]
