# Supplementary material for: A Novel Role for Banana MaASR in the Regulation of Flowering Time in Transgenic Arabidopsis
Source: PLoS One. 2016 Aug 3;11(8):e0160690. doi: 10.1371/journal.pone.0160690 (PMC4972433; doi:10.1371/journal.pone.0160690)
Supplement: S1 Table — (DOC) [file pone.0160690.s007.doc]

**Table S1 Primers used in the study**

| Gene Name | Sequence(5′ to 3′) |
| --- | --- |
| *MaASR* | F: agaagcatcaccatcatctc; R: caagcatcccacactcaacac |
| *Ptr* | F:CTCCGAGATCTGGACGAGC; R: TAATACGACTCACTCACTATAGGG |
| *MaACTIN* | F: cgaggctcaatcaaaga; R: accagcaaggtccaaac |
| *MaUBQ* | F: GGCACCACAAACAACACAGG; R: AGACGAGCAAGGCTTCCATT |
| *AtACTIN* | F: CCAACAGAGAGAAGATGA; R: ATGTCTCTTACAATTTCCCG |
| *AtUBQ* | F: GACGCTTCATCTCGTCC; R: CCACAGGTTGCGTTAG |
| *AtFCA* | F: CGGATAGAGCCATCAGAGC; R: AACAAACCCACATCCACGA |
| *AtFLK* | F: TCGCAAGATGACCTACAA; R: GAACCAGCATACGGAATA |
| *AtFRI* | F: ACTACCAGGATGGCAGAT; R: TTGAGCGATGAGGAAAA |
| *AtGAI* | F: AAATGGCTGATGTTGCTC; R: AGATCGTACTCGGCGTTA |
| *AtLFY* | F: GATGCGACTTGGTGGTTT; R: GCAATCGTCTCCGTTCAG |
| *AtRGL1* | F: GTTCACGGAATCGCTACA; R: GCATAAAGTGCCAACAACA |
| *AtVRN1* | F: TTTTATTCTCCGTCGTCGTT; R: ACAAACTCTTGCCAACCAT |
| *AtFLC* | F: CGGTTGAAATCAAAATCCAAAACA; R: CACACGAATAAGGTACAAAGTTCATC |
| *AtFVE* | F: ATGCAGATACTAACTGGGCACCAA; R: AATCTGTCCCAATCGTTGTGATGT |
| *AtSOC1* | F: ACCATAGATCGTTATCTGAGGCAT; R: GAAGAACAAGGTAACCCAATGAAC |
| *AtCol1* | F: AGACCAACCACCTTACCCT; R: GCCATAGCAGCATCAGAAT |
| *AtCol2* | F: TAACAGTCCAACACCCAAGA; R: TCGCCATAGGAGTTTAGAAG |
| *AtNAP* | F: AACTTCCCAATCTACCCT; R: GTGAATGGCTTTGTCTGT |
| *AtTCH2* | F: AATCGGAGGAGGAGGTAA; R: TAATCGCCGTCACTAAAA |
| *AtSEP3* | F: CGTTATGACGCCTTACAG; R: TCTTGGTTAGGGTTCAGC |
| *AtCol9* | F: TCACATTCAGTAACCCGTAA; R: CACTTGAAACTGCCCATA |
| *AtCO* | F: CCAAAGGGACAGTAGAGC; R: TTGCCACAGGAGTATCAG |
| *AtELF3* | F: AGAAAGAGCAGGCAAGGG; R: TGGCAGCATTCTCACTCG |
| *AtELF4* | F: AGGAACGGCGAGACGAAA; R: GTGAAAGCCCGACGAGAA |
| *AtNGA1* | F: TCCCTGACCATCCTCATTTC; R: GCCGCATTCCATATCAACTC |
| *AtMAF5* | F: GCAAACTCTACAACTCCTCCTC; R: GGTTCTTCACAAGCTCCATC |
