# Supplementary material for: A Novel Role for Banana MaASR in the Regulation of Flowering Time in Transgenic Arabidopsis
Source: PLoS One. 2016 Aug 3;11(8):e0160690. doi: 10.1371/journal.pone.0160690 (PMC4972433; doi:10.1371/journal.pone.0160690)
Supplement: S2 Table — (DOC) [file pone.0160690.s008.doc]

**Table S2 Statistical plant numbers listed in this study**

| Line name | 1 | 2 | 3 | 4 | 5 |
| --- | --- | --- | --- | --- | --- |
| WT | 36 | 68 | 126 | 69 | 45 |
| L14 | 36 | 59 | 118 | 70 | 48 |
| L38 | 72 | 60 | 121 | 72 | 42 |
